# Monitoring Indian “Superfood” *Moringa oleifera* Lam. – species-specific PCR-fingerprint-based authentication for more consumer safety

**DOI:** 10.1038/s41538-024-00264-z

**Published:** 2024-04-13

**Authors:** Sascha Wetters, Vaidurya Sahi, Lena Brosche, Annette Häser, Peter Nick

**Affiliations:** 1 Department of Molecular Cell Biology, Joseph Gottlieb Kölreuter Institute of Plant Science (JKIP), Karlsruhe Institute of Technology, Fritz‑Haber‑Weg 4, 76131 Karlsruhe, Germany; 2 Department of Genetics and Plant Breeding, Sam Higginbottom University of Agriculture, Technology and Sciences, Prayagraj, 211007 UP India

**Keywords:** Plant sciences, Molecular biology

## Abstract

*Moringa oleifera* Lam. has become one of the major new superfoods commonly available in the aisles of bio*-*shops and health-food sections in supermarkets of North America and Europe. While most of these products appear under the generic and scientifically inconclusive term “Moringa”, the European Union, so far, has allowed commercialisation for the use in food and feed for *M. oleifera* only. *M. oleifera* is indigenous to India and South Asia, but large-scale cultivation of this species has spread to the tropical regions on all continents, with a strong focus on Africa, leading to a high risk of admixture with species like *M. stenopetala* (Baker f.) Cufod. that is native to Africa. In the present study, we have characterised six species of *Moringa* in order to develop a simple and robust authentication method for commercial products. While the plants can be discriminated based on the pinnation of the leaves, this does not work for processed samples. As alternative, we use the plastidic markers *psbA-trnH* igs and *ycf1b* to discern different species of *Moringa* and develop a diagnostic duplex-PCR that clearly differentiates *M. oleifera* from other *Moringa* species. This DNA-based diagnostic assay that does not rely on sequencing was validated with commercial products of “Moringa” (including teas, powders, or capsules). Our method provides a robust assay to detect adulterations, which are economically profitable for costly superfood products such as “Moringa”.

## Introduction

An increasing awareness for health in wealthy, but ageing societies, and the trend to boost economic performance by self-optimisation has led to a boom of a new food category commonly known as “superfoods”. The Oxford dictionary defines superfood as “*A nutrient-rich food considered to be especially beneficial for health and well-being”*. In a more recent definition^1^, superfoods are described as “*an increasingly significant category of health foods that are celebrated for their supposed extraordinary nutritional and/or medicinal properties, their histories of traditional use by ancient or indigenous communities, and their ‘natural’ and ‘authentic’ qualities”*. Six main characteristics have been attributed to superfoods: being whole, conferring specific nutritive values, transporting ethical value, being of preventive rather than curative nature, relating to neoliberal values, and also rating high in terms of social parameters, such as sustainability or fair trading^2^. Superfoods can be very diverse ranging from the prokaryote *Spirulina*^3^ to plants, and meanwhile include new trends using insects^4^. Industrialised countries, such as the US or Germany have become major importers for food and health products labelled as superfoods^5^. Actually, superfood not only relates to exotic products, such as “Chia” or “Goji”, but also includes autochthonous plants like the cabbage *Brassica oleracea* L., which in Germany has been in use for centuries^6^. A recent report lists 217 species as superfoods (Butterworth et al. ^2^). Many of those are rooted in traditional medicinal systems, such as Ayurveda or Traditional Chinese Medicine, where functional food is a central element of preventive care. This means that these products often harbour pharmacologically active compounds, creating issues for consumer protection Therefore such plant products are subject to legislation, such as the Novel-Food regulation, where products not in use prior to 1997, can be traded only, if they are found on a list of permitted species^7^.

With a current global market volume of 5800 million US$ estimated to double during the next five years^8^, *Moringa* has to be considered as one of the emerging superfoods in industrialised countries^9–12^. According to the aforementioned six characteristics, *Moringa* clearly qualifies as superfood which is reflected in price, marketing strategies, and consumer expectations. While not traditionally used in the West, one species of this genus, *Moringa oleifera*, is explicitly exempted from the Novel Food regulation^7^, since there are records that it had been used prior to the introduction of this legal classification.

However, the genus *Moringa* (belonging to the family of the Moringaceae, a sister clade of the Brassicaceae) comprises 13 different species from tropical and subtropical climates. Although small, this genus presents considerable morphological variability: The plants can be anything from small shrubs to massive trees^13^, and the flowers range from radial to bilateral symmetric^14^. *Moringa*, originating in the seasonally dry tropics of Africa, Asia, and Madagascar, can be divided into three life forms^15^, which is also reflected by geographical distribution: The “bottle trees” (e.g *M. stenopetala*) have massive water-bearing strains with fleshy roots and radial-symmetrical flowers, and occur in Africa and Madagascar. The “slender trees” (e.g., *M. oleifera*) have a slender trunk and tough, fibrous roots, as well as bilaterally symmetrical flowers. They are predominantly found in South and South-East Asia. The last and most species-rich “tuberous group” (e.g., *M. pygmaea* (Forssk.) Fiori) consists of small trees or shrubs from North-East Africa, which have fleshy and water-bearing roots as well as bilaterally symmetrical flowers.

Within this genus, *Moringa oleifera* is the economically most relevant species, native to the sub-Himalayan regions of South Asia and characterised by slender trunks at maturity and roots with a bark that is smoother and more fragile than that of the stem^16^. This fast-growing tree, also known as Horseradish Tree, has been domesticated by the ancient Hindus, but was also known to the Egyptians, Greeks, and Romans^17^. *Moringa oleifera* has been in use for more than 4.000 years in human and animal nutrition, as well as for traditional medicine^18–20^. Different parts of the *Moringa oleifera* tree (*Meerrettichbaum* in German, Horseradish Tree in English, *Shigru* or *Shohanjanah* in Sanskrit, *Murunkai* in Tamil, *Sahijan* in Hindi) are therapeutically used in different ways as mentioned in the Ayurveda^21^. The superfood quality of *Moringa oleifera* is often advertised to be linked with a high proportion of important minerals, vitamins, beta-carotene, amino acids, and various phenols, such as quercetin or kaempferol, or with hormones such as the cytokinin zeatin (reviewed in^22,23^). *Moringa* is claimed to contain more iron than spinach, more vitamin A than carrot, more vitamin C than oranges, more calcium than milk, more potassium than bananas, and more protein than yogurt^24,25^. While these nutrients are often missing among populations of underdeveloped or developing countries, these features are not very specific and would hold true for almost any vegetable or fruit. Moreover, there is no reason to believe that the typical consumer in industrialised countries living under conditions of complete affluence would rely on a superfood for sufficient uptake of proteins or vitamins. Thus, there might be components more specific that render *M. oleifera* into a superfood. The fact that the leaves or the fruit of the *Moringa* tree are used against various diseases such as malaria, arthritis, skin disorders or hypertension, or to strengthen the immune system^26^, indicates the presence of specific active compounds. The recent finding of specific glucosinolate species that differ in profile and content between different species of *Moringa* and show different levels of cytoprotectivity in different mammalian cell lines support the presence of such specific compounds^27^.

Currently, the European market is flooded with numerous *Moringa* products, commonly found on shelves even of supermarkets. The products are available in the form of tea mixtures, dried leaves, powdered leaves, smoothies, capsules, and unprocessed fruits of *Moringa*. According to the Novel Food regulation^7^, *Moringa oleifera* is the only species admitted for the European market. The European Food Safety Authority has recently (2019) turned down the attempt to introduce the African species *M. stenopetala*, due to possible effects on thyroid metabolism^28–30^. While *M. oleifera* imports reach the EU mostly from India, the potential of this rapidly growing market has progressively attracted producers in Africa^31,32^. As a result, *M. stenopetala* or *M. drouhardii* (Baker f.) Cufod., whose leaves resemble those of *M. oleifera* but differ in biochemical profiles^33^, especially with respect to the bioactive glucosinolates^27^, could turn into major contaminants in imports originating from Africa and declared as *M. oleifera*. In many cases, the plain declaration as “*Moringa*” can be quite misleading in view of the 13 species belonging to this genus. The rapidly growing consumer demand, the substantial price of these products, and the ignorance of biological diversity creates ideal conditions for the usage of counterfeit or surrogate ingredients. The fact that *M. stenopetala* is also in common use as food supplement outside of Europe and readily available all over Africa^34^ accentuates the problem even further.

The presence of potential counterfeit species that are often much cheaper than the real plant product calls for efficient systems to protect consumers from possibly toxic plants. However, while many countries routinely conduct authentication on the base of well-established pharmacopeias, the case of novel plant products, such as *Moringa*, poses huge challenges. The classical microscopic diagnostics using discriminative anatomical or morphological features is often not possible in case of highly processed products such as *Moringa* powders or smoothies. Here, the morphological characterisation needs support from diagnostic DNA markers^35,36^.

The vision of a “DNA barcode”, where each plant, in analogy to a product in the supermarket, can be unequivocally identified, has stimulated an intense search for markers that can be amplified by PCR using universal primers, can be sequenced readily, and are sufficiently informative to differentiate between different species^37^. Plastidic markers are widely used, because cpDNA exists in many copies per cell, such that they are readily amplified. Moreover, inheritance of plastidic genes is preferentially maternal, such that it is easier to infer haplotypes. The quite variable *psbA-trnH* intergenic spacer region between the highly conserved *psbA* and *trnH* genes^38^ can differentiate in many cases even between species within a genus. The more recently introduced plastidic marker *ycf1b* can provide an even superior level of discriminative power^39^. Among nuclear markers, mostly the internal transcribed spacer (ITS), in particular the section ITS2^40^, has been used for DNA barcoding.

These barcodes are useful to infer phylogenetic relationships between species. For the pragmatic purpose to discriminate a declared species against surrogates, it is desirable to draw upon fingerprinting approaches that do not rely on (more time consuming) sequencing, alignments, and similarity searches. Such fingerprinting strategies make use of informative Single Nucleotide Polymorphisms (SNPs) or indels in these barcoding markers. For this purpose, restriction length polymorphism (RFLP) has been successfully employed^41–43^. Alternatively, a specifically designed duplex PCR can discriminate species pairs of interest. By introducing destabilising mutations into the 3’-end of an additional primer that is annealing to the informative region, binding occurs only for one of the species, while even a single SNP in the other species, in combination with the de-stabilisation, will eliminate binding. This so-called Amplification Refractory Mutation System (ARMS) will then lead to a diagnostic side band for the first species, but not for the other. This robust molecular diagnostic tool has been successfully used to detect adulteration of plant products^42–46^.

The current study deals with the use of morphological and molecular markers to authenticate *Moringa* products as contribution to consumer safety. Using reference plants that had been carefully authenticated by classical taxonomy, we explore the limits of microscopic analysis, supporting the need for DNA-based assays. We then develop a diagnostic assay based upon an ARMS strategy *ycf1b* as molecular marker and show that this allows to differentiate between *Moringa* species from Asia and Africa. In addition, we present a complementary assay that allows sorting out the canonical species *M. oleifera* from all other known species of *Moringa*.

## Results

### *Moringa* species can be differentiated based on leaf morphology and anatomy

**Fig. 1:**
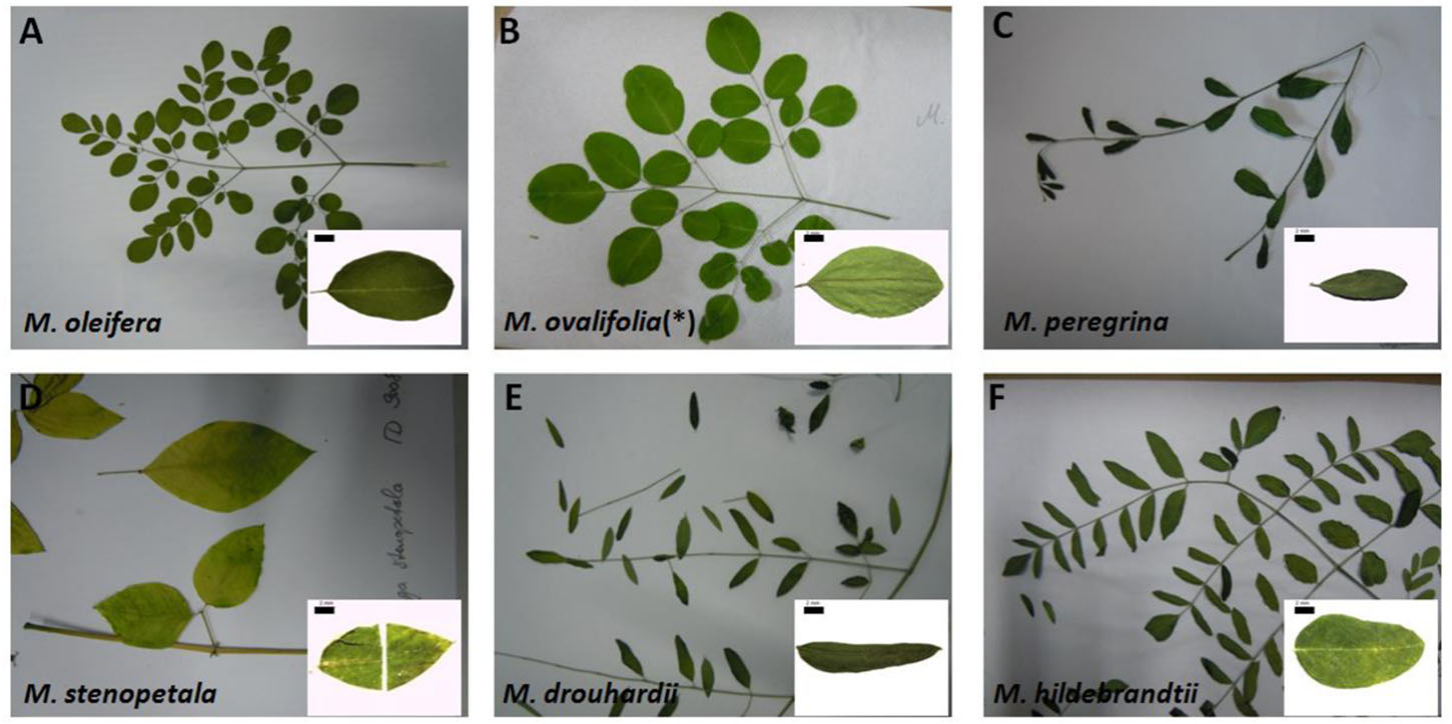
Comparison of leaf appearance of *Moringa* species of our study. Images of single leaflets (inset) were recorded using a stereomicroscope at a magnification of 6.3x (scale bar in inset is 2 mm). (*) received as *M. ovalifolia*, by re-evaluation determined as *M. oleifera*. **A** *Moringa oleifera*, (**B**) *M. ovalifolia* (*), (**C**) *M. peregrina*, (**D**) *M. stenopetala*, (**E**) *M. drouhardii and* (**F**) *M. hildebrandtii*.

Since commercial products of *Moringa* derive from leaves, we first searched for morphological or anatomical traits that might serve for diagnostic differentiation in commercial samples. Using leaves from authenticated reference plants, we searched for differential features, but this turned out difficult, since all leaves are pinnate (Fig. 1). However, a closer look showed clear differences in leaflet shape. The tripinnate leaves of *M. oleifera* are ovoid and asymmetric with respect to their long axis (Fig. 1A). If the leaf were approximated by a rhomboid, the cross axis of this rhomboid would be clearly shifted towards the tip, such that the basal part of the rhomboid would appear narrower, while its apical part would appear broader. This contrasts clearly with the pointed leaves of *M. stenopetala* (Fig. 1D) or M. *drouhardii* (Fig. 1E). The leaves of *M. hildebrandtii*, reached their widest cross axis in the basal half, such that the approximated rhomboid would here appear narrower at the tip and wider at the base (Fig. 1F). For a specimen declared as *M. ovalifolia*, the leaf tips are replaced by a characteristic notch, not seen in any of the other inspected species. However, when this accession was flowering, it turned out to be *M. oleifera* (Fig. 1B). Based on shape and size, the leaflets of *M. peregrina* (Fig. 1C) were also displaying their widest axis shifted to the apex and resembled in that respect the leaflets from *M. oleifera* (Fig. 1A). However, the two species could be easily discriminated based on their overall shape – leaflets in *M. oleifera* were distinctly broader as compared to the leaves of *M. peregrina*.

Since leaf shape, due to processing, is often not preserved in commercial samples declared as Moringa, we were searching for anatomical traits that could be employed for microscopic diagnostics. Epidermal pavement cells, often useful as diagnostic marker, differed in the depth of lobing, from almost polygonal shapes in *M. hildebrandtii* till strongly interdigitated cells in *M. oleifera* (Supplementary Fig. 1). However, the differences between the species were not conspicuous enough to serve as trait in microscopic diagnostics, especially in leaf fragments of commercial products.

**Fig. 2:**
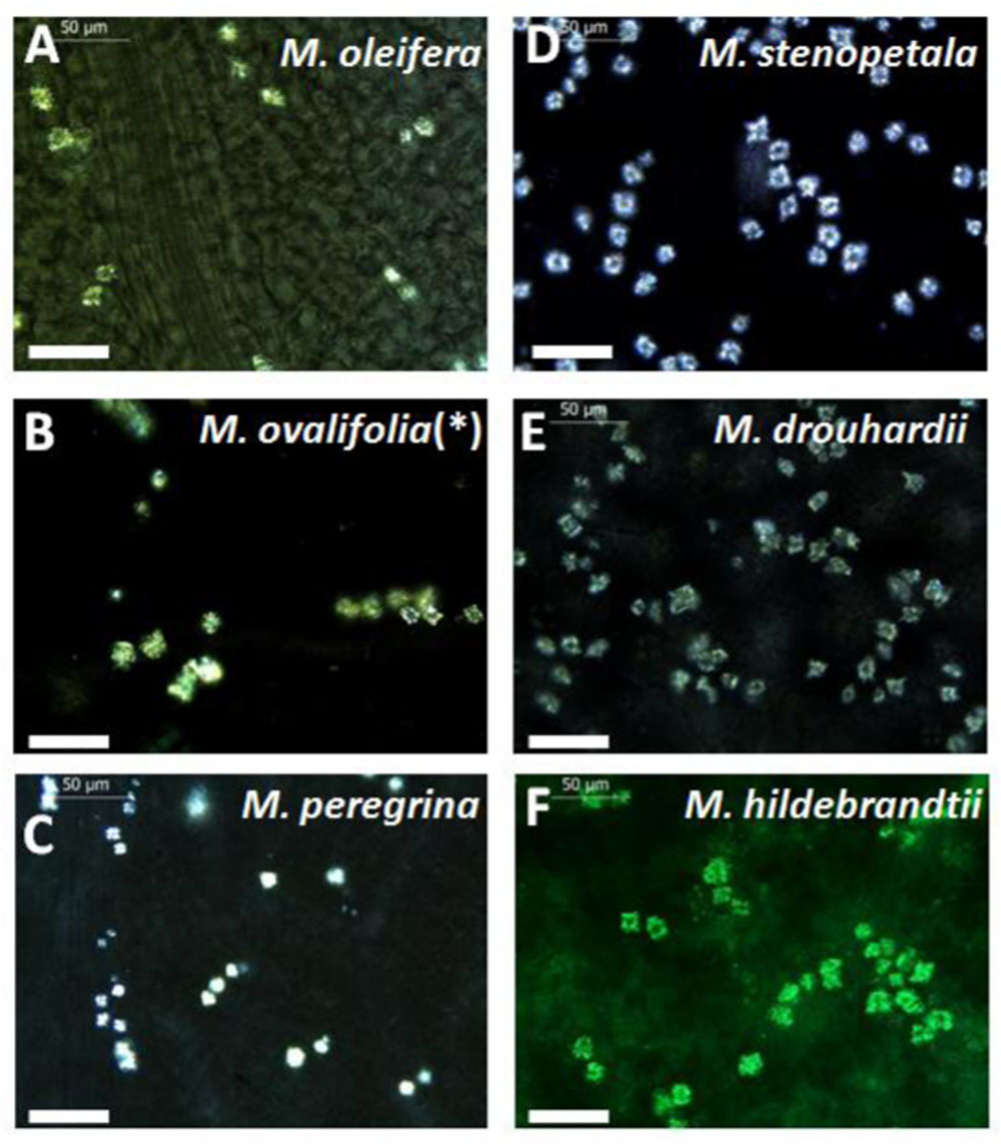
Cells with calcium oxalate glands in the spongy parenchyma of *Moringa* species. Calcium oxalate crystals were visualised in the spongy parenchyma layers of the leaf using polarised light. (scale bar 50 μm). (*)received as *M. ovalifolia*, by reevaluation determined as *M. oleifera*. **A** *Moringa oleifera*, (**B**) *M. ovalifolia*(*), (**C**) *M. peregrina*, (**D**) *M. stenopetala*, (**E**) *M. drouhardii and* (**F**) *M. hildebrandtii*.

A second trait were idioblasts in the spongy parenchyma, harbouring calcium oxalate crystals that became visible by polarisation microscopy (Fig. 2). However, these crystals were found in all reference species in similar size and morphology, such that also this trait did not qualify as criterion to discern *M. oleifera* from other *Moringa* species.

### Morphological differentiation in commercial samples of *Moringa* is not feasible

**Fig. 3:**
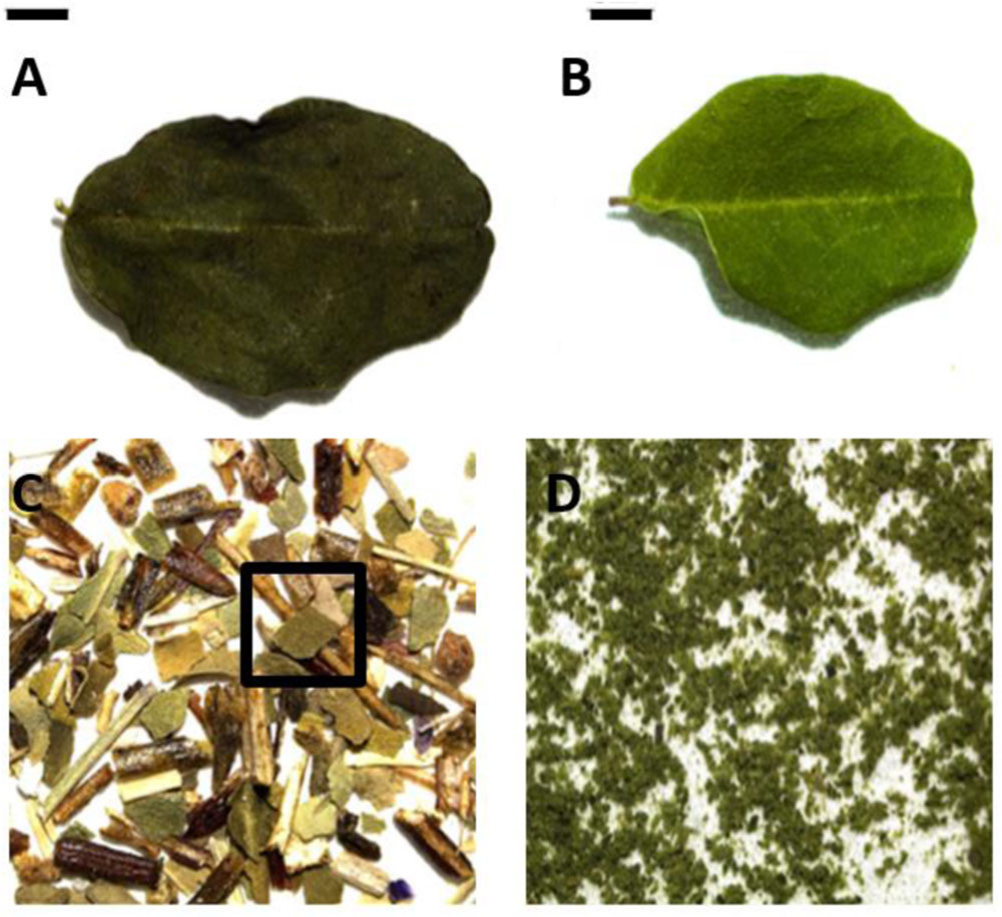
Macroscopic analysis of commercial products containing *Moringa.* Commercial products come in different stages of processing, as complete leafs (**A**, **B**), as tea with *Moringa* leafs (black square) among other plants (**C**) and as powder (**D**). Scale bar is 2 mm.

When we tried to apply the morphological and anatomical characteristics seen in the reference plants (Figs. 1, 2) to commercial products declared as “Moringa”, it became rapidly clear that this is not feasible. Only a few products contained entire leaves of *Moringa* which would allow recognising them as *M. oleifera* due to the characteristic shape of the leaflets (Fig. 3A, B), while the majority of commercial samples was processed to a degree that would not support microscopic diagnostics. Either was *Moringa* only a component of a tea mixture, and present only in fragmented form, such that it even was difficult to define adaxial or abaxial leaf face (Fig. 3C), or, even worse, the leaves were ground to a powder to be used in smoothies and the like (Fig. 3D). To discern any diagnostic features here is even impossible to the expert of microscopic food diagnostics, not to speak for a person without respective training.

As microscopic diagnostics comes to a limit here, we have explored molecular markers to reliably identify the Superfood *M. oleifera*. In order to clear the phylogenetic relationship of the different *Moringa* species and the commercial products used in this study, we used the plastidic *psbA-trnH* igs and *ycf1b* marker.

### The *psbA-trnH* igs barcode can differentiate *M. oleifera* but is not suited for fingerprinting assays

The multiple sequence alignment of the different *Moringa* individuals based on the *psbA-trnH* igs revealed different nucleotide substitutions and deletions.For the closely related *M. peregrina* (from the Arab peninsula) and *M. oleifera* (from India), we found substitutions at positions 320 bp (C) and 381 bp (A) in the alignment, in addition to a 9-bp insertion in *M. peregrina* that is absent in *M. oleifera*. Also, sequences we got for the African species *M. hildebrandtii*, *M. drouhardii*, *M. stenopetala* and *M. ovalifolia* (complemented with sequences from GenBank) harboured deletions and several nucleotide substitutions. The phylogenetic tree inferred from these sequences (Supplementary Fig. 2) revealed an African cluster, containing *M. drouhardii*, *M. hildebrandtii*, *M. ovalifolia* and *M. stenopetala*, which is sister to the two accessions of *M. peregrina*. All *M. oleifera* reference plants as well as the commercial products are clearly separated from this African / Arabian cluster. This barcoding marker region demonstrates that all of the commercial products are *M. oleifera*. Moreover, *peregrina* is closer to the African *Moringa* species, rather than to *M. oleifera*. However, the split of those two clusters is not supported by significant bootstrap values. Although the *psbA-trnH* igs differentiates *Moringa oleifera* from all other species, it was not possible to derive a sequence-free fingerprinting assay. The sequence differences did not concern any of the known restriction sites, such that a RFLP strategy was not feasible. Likewise, the differences were located in AT-rich regions (AT content was generally very high with 74,9% on average), such that an ARMS strategy was not feasible either, because it was not possible to design diagnostic primers with sufficient discriminative power. Thus, a different barcoding marker region was needed to achieve the goal to differentiate between *M. oleifera* and other species of *Moringa*.

### The *ycf1b* barcode provides an alternative that allows for fingerprinting assays

**Fig. 4:**
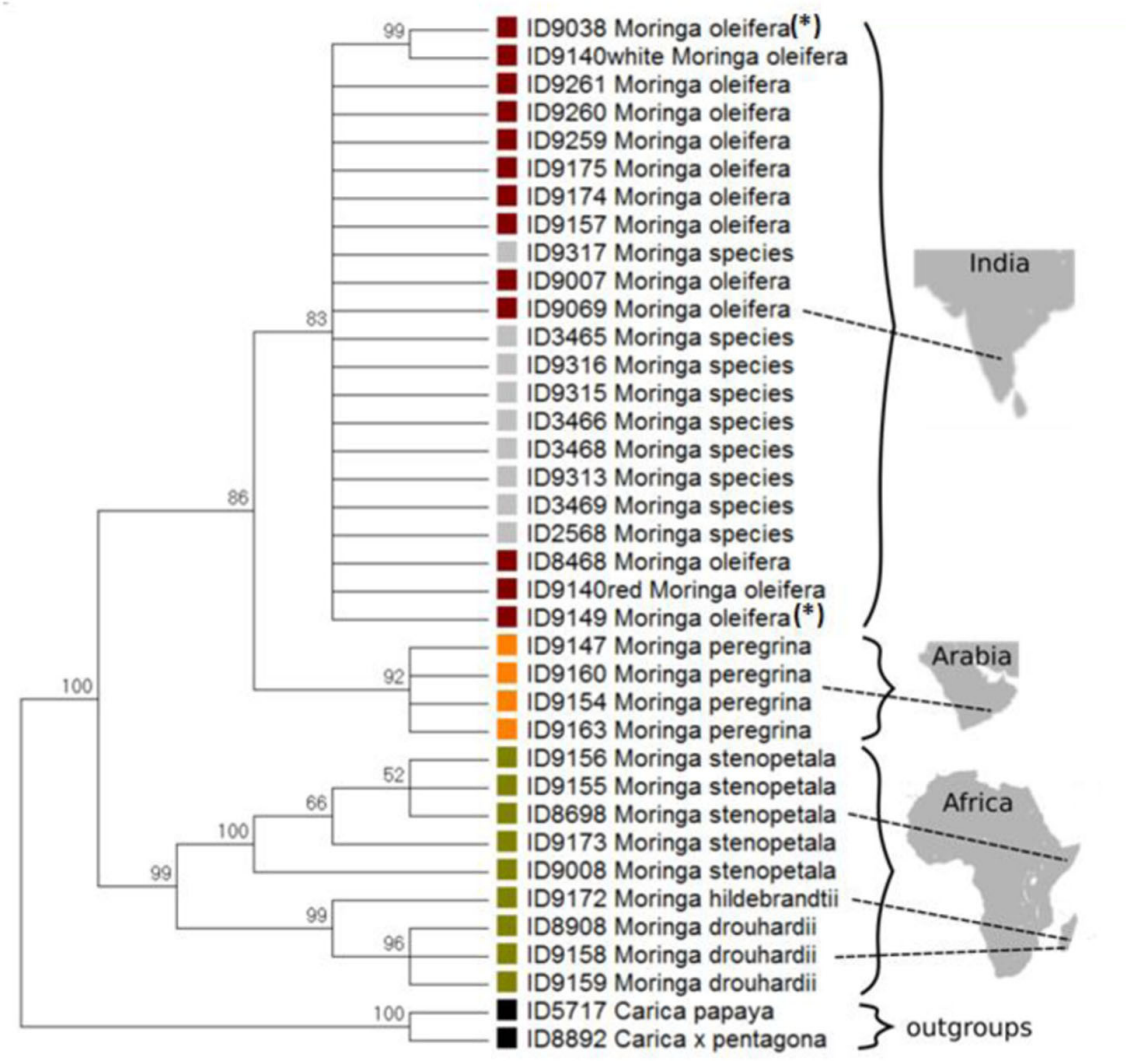
Neighbour joining phylogenetic tree based on the plastidic ycf1b marker. The reference plants are represented as coloured squares, with red for Indian *M. oleifera*, yellow for African *Moringa* species (including *M. hildebrandtii*, *M. stenopetala* and *M. drouhardii*) and orange for *M. peregrina* individuals. Commercial products are displayed with a grey square. The outgroups *Carica papaya* and *C. pentagona* are represented as black squares. The internal ID of the Botanical Garden of the KIT (see also Table 1) for the ycf1b fragments are given next to the species name. The numbers on the branches indicate the reliability of the clusters by means of 1000 bootstrap replications. The geographic regions are not drawn by scale. (*) received as *M. ovalifolia*, by re-evaluation determined as *M. oleifera*.

Unlike the intergenic spacer region of *psbA-trnH*, the *ycf1b* marker region codes for an essential plastidic protein, such that deletions (often resulting in frameshifts) do not occur. We explored the suitability of this marker to discriminate the individual Moringa species and to design a fingerprinting assay that would be able to delineate *M. oleifera* from other species of the genus. In fact, the tree (Fig. 4) inferred from the multiple alignment trimmed to 722 basepairs displays a statistically significant supported split of Asian (*M. oleifera* and *M. peregrina*) from African (*M. drouhardii*, *M. hildebrandtii*, *M. stenopetala*) *Moringa* species. For the latter, a separation of the endemic Madagascan species *M. drouhardii* and *M. hildebrandtii* from *M. stenopetala* originating from the Horn of Africa is supported by high bootstrap values (>99%). Likewise, all accessions of the morphological closely related Asian species *M. oleifera* and *M. peregrina* were clearly distinguished by the *ycf1b* marker (with bootstrap values > 90%). The only exception was the congruence of two accessions declared as *M. ovalifolia* with *M. oleifera*. However, as already pointed out above, based on their floral traits, these accessions could be later identified as *M. oleifera* as well, such that their location in the *M. oleifera* clade became perfectly consistent. Overall, the *ycf1b* marker allowed to distinguish all individual species of the genus.

The tested commercial products of *Moringa*, including teas, powders, and capsules, (with their often- indiscriminate labelling) collectively clustered with the reference plants of *M. oleifera*. Two commercial products that were advertised as *M. ovalifolia* could be classified as *M. oleifera* by clustering with respective samples.

The *ycf1b* alignment exhibited a few substitutions that were private for *M. oleifera* and even differed from the closely related Asian species *M. peregrina*. Fortunately, these substitutions were located in a region of favourable GC content. This encouraged us to venture for an easy one-step discrimination tool to distinguish *M. oleifera* from all other *Moringa* species as diagnostic assay that can also be applied for the identification of commercial *Moringa* products.

### A simple and robust ARMS approach for the identification of “Superfood” *Moringa oleifera*

The aforementioned nucleotide substitution in the plastidic *ycf1b* sequences of different *Moringa* species was used to design ARMS primers that could be utilised in a multiplex-PCRs to distinguish *M. oleifera* from adulteration by other *Moringa* species. This identification is based on the co-amplification of a second (diagnostic) band with a size of ~650 bp that in case of *M. oleifera* appears in addition to the full-length *ycf1b* fragment (size ~950 bp) upon agarose gel electrophoresis.

**Fig. 5:**
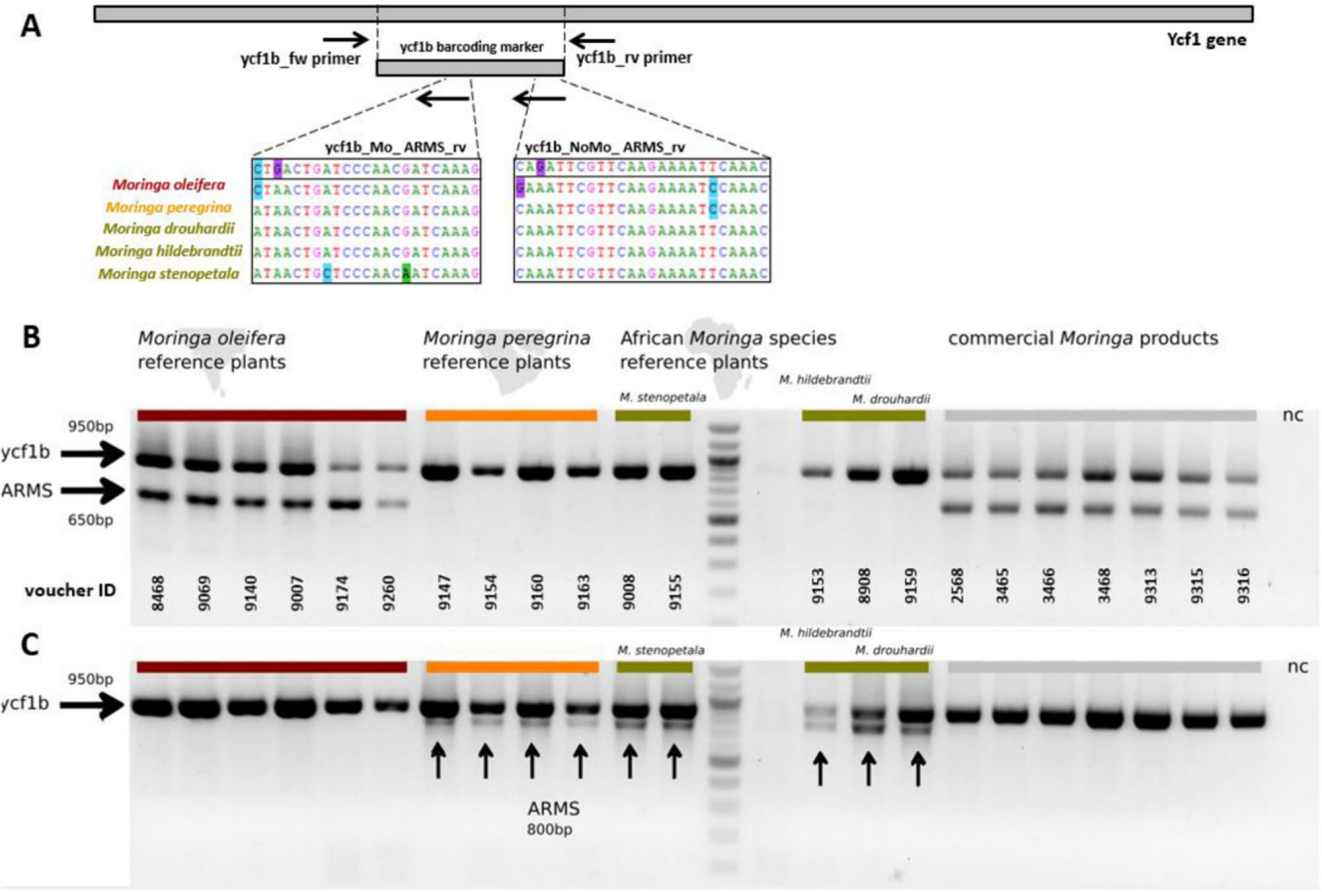
ycf1b-based ARMS diagnosis for differentiating between *Moringa oleifera* and other *Moringa* species. The commercial products and the reference plants are highlighted in different colours, according to the phylogenetic tree. **A** Schematic illustration of the ycf1 region and orientation of primers used in this study. The decisive nucleotide substitutions were detected in the ycf1b region of *Moringa*. Sequence cutouts of different *Moringa* species and the primer sequences are highlighted. **B** Duplex-PCR with ycf1b fw / rv and the additional diagnostic ycf1b_Mo_ARMS_rv primer. All samples display a ycf1b control band with a fragment size of around 950 basepairs. An additional diagnostic ARMS band with a fragment size of 600 basepairs is clearly visible in *M. oleifera* reference plants (labelled red) and the commercial *Moringa* products (labelled grey). This specific *M. oleifera* band is completely absent in African *M. stenopetala*, *M. hildebrandtii* and *M. drouhardii* (labelled yellow) and Arabic *M. peregrina* (labelled orange). **C** Duplex-PCR with ycf1b fw / rv and the additional diagnostic ycf1b_NoMo_ARMS_rv primer. All samples display a ycf1b control band with a fragment size of around 950 basepairs. An additional diagnostic ARMS band with a fragment size of 800 basepairs is present in all African individuals (labelled yellow) and also the Arabic *M. peregrina* (labelled orange). This specific *Moringa* band (excluding *M. oleifera*) is absent in *M. oleifera* and all *Moringa* commercial products.

For this purpose, two diagnostic primers were developed that were complementary in their target (see schematic depiction in Fig. 5A, and Table 3): Using the diagnostic ycf1b_Mo_ARMS_rv primer in addition to the universal *ycf1b* primer pair, the expected diagnostic band was present in all accessions of *M. oleifera*, while it was absent in all accessions from other *Moringa* species (Fig. 5B). Conversely, the usage of diagnostic ycf1b_NoMo_ARMS_rv primer combined with the *ycf1b* primer pair produced the full-length band along with the diagnostic *ycf1b* fragment in all *Moringa* species, but *M. oleifera* (Fig. 5C). In all tested commercial *Moringa* products, the presence of the diagnostic side band for ycf1b_Mo_ARMS_rv, and its absence for ycf1b_NoMo_ARMS_rv reported that these products contained indeed *M. oleifera*, and not a different *Moringa* species.

## Discussion

The current work was motivated by the need for a diagnostic tool to authenticate *Moringa oleifera* as only species admitted for trade in Europe and to discern it from other species of the genus that are common in Africa, emerging as one of the major producers for “Superfood Moringa”. Using a set of authenticated reference plants, we show that all species of the genus can be distinguished from their leaf morphology. However, we demonstrate that microscopic diagnostics of processed samples is not feasible. We explore then the possibility of genetic barcodes as tools. While both tested markers, *psbA-trnH* igs and *ycf1b* allow to infer phylogenies with sufficient resolution to discern *M. oleifera* from its possible surrogates, only the *ycf1b* marker allows for a sequencing-free fingerprinting strategy using a diagnostic duplex PCR. We validate this assay with commercial samples traded in Germany and show that all of these samples are from *M. oleifera* as they should be. In addition to its practical application, this work leads to a couple of questions that will be discussed below: Why should consumers be protected at all against adulteration of “Superfood Moringa” by species other than *M. oleifera*? What are the requirements for a marker to be suitable for authentication by sequencing free DNA fingerprinting, what are the limitations of the current approach and what possibilities exist to address those in future studies? What is the potential of this assay on the background of the current dynamics in the market for Moringa?

With novelty comes uncertainty. For example, as soon as tea was introduced into Europe, its adulterations started^47^. With adulteration came a need to authenticate products. Whether adulteration is accidental (for instance, due to ambiguous vernacular nomenclature), or whether it is deliberate^48^, it will at the end result in consumer-deception. Globalisation means also that products enter new markets, often over great geographical distance. The trade of plant products in general, and the emerging trade of plant superfoods, such as “Moringa” very saliently reflect this conflict between globalisation and consumer safety. The European Union has tried to mitigate this by legislative measures, such as the Novel Food regulation^7^, which confines commercialisation of “Moringa” to the species *Moringa oleifera*. While Africa as emerging producer does also export *Moringa oleifera*, there is a history of use of other *Moringa* species. “Moringa” has turned into a high-priced product, and since the knowledge, and the sensitivity for the taxonomic differences is not to be expected in growers and traders. This situation increases the risk for adulteration or admixture of the other *Moringa* species in commercial products exported to Europe. If the claimed health benefit of *Moringa oleifera* were linked to relatively unspecific traits such as content of carotinoids, vitamin C, calcium, potassium, or protein^24,25^, such adulterations would be more or less irrelevant for the consumer. It is not very likely to assume that the general metabolism of closely related species should be principally different. However, it is becoming progressively clear that the health benefits ascribed to *Moringa* are linked with specific secondary compounds, especially the glucosinolates^27^, whereby glucomoringin (4-[(α-L-rhamnosyloxy)-benzyl glucosinolate]) is the lead compound in the canonical *M. oleifera*^49,50^.

In fact, the two main cultivated species of genus *Moringa* (*M. oleifera* and *M. stenopetala*) display considerable differences in abundance of glucosinolates^51,52^. Additionally, the composition of glucosinolates differs between different species of the genus *Moringa*, but even on the intra-species level between domesticated versus wild individuals of *M. oleifera*^27^. Upon ingestion, glucosinolates are cleaved by myrosinase released from the wounded plant tissue to release different isothiocyanate aglycons. Moringin, the aglycon of glucomoringin, this is the moringin, exerts very specific responses, for instance such as the activation of the somatosensory receptor channel TRPA1, which may account for the analgesic effect of *Moringa oleifera*^53^. A comparative study in *M. oleifera* and *M. stenopetala* collected from different sites across West Africa showed that the glucomoringine contents were much lower in the African species^51^. Thus, adulteration of *M. oleifera* by *M. stenopetala* mean that the consumer is confronted with a product where the main bioactive compound is depleted.

Microscopic diagnostics based on morphological or anatomical characteristics can be a low-cost and robust approach to authenticate commercial samples^36^. However, it is only as reliable as the references used to calibrate the assignments of observed trait to a given species. The use of reference material that had been taxonomically authenticated is mandatory^54^ and represents a quality criterion for any authentication, independent of the method. In the current study we used reference plants of *Moringa* species cultivated in the Botanical Garden of the KIT. Leaf morphology and pinnation are helpful taxonomic markers for *Moringa* species, but show a certain degree of intraspecific variation^55^. Unfortunately, this trait is not available in most commercial products such as herbal teas and supplements of *Moringa* that come either in mixed, crushed, or powdered form. Thus, analysis of the cellular details in leaf fragments remains as last resort for microscopic diagnostics. In this context, epidermal cells, and their derivatives such as guard cells and their relative size over the subtending palisade parenchyma can sometimes allow to separate even species from the same genus, as shown during a systematic study on different species of *Ocimum*^56^. While we could identify anatomical traits that allowed to discern different species based on intact leaf material, such as the depth of pavement lobing (Supplementary Fig. 1), it became rapidly clear that such traits would not work in the strongly processed commercial samples, where it is often impossible to tell, whether a given fragment stems from the adaxial or the abaxial side of the leave, such that the quantitative differences in lobing degree between the two faces of a leave would mask potential differences between species. Thus, there is no alternative to molecular authentication.

For the present study, we initially applied the commonly used the plastidic *psbA-trnH* igs barcoding marker, because it is informative and versatile with fragment lengths that vary from 152 to 851 basepairs in eudicots, with an average length of 357 basepairs^57^. While there is a notable difference in fragment length with around 550 basepairs in African species as compared to 450 basepairs in Asian and Arabic *Moringa* species, this length polymorphism alone is not sufficient to assign a given specimen based on the electrophorogram if one cannot rely on validated reference plants from both regions. However, the sequence amplified for this marker allows to discriminate very clearly *M. oleifera* reference plants and commercial products from African species of *Moringa* (Supplementary Fig. 2). Thus, a fingerprinting assay should be feasible. However, sequence variation is not the only factor –all candidate regions are so rich in AT that it is impossible to design a sequence-free identification ARMS strategy. We were testing, therefore, the plastidic *ycf1* gene. This marker meets central criteria for barcoding markers, such as universality in plants, suitable length, and abundance of informative sites, such that it had been proposed “the most promising plastid DNA barcode of land plants“^39^. In fact, this marker not only resolved individual species of *Moringa* at with sufficient bootstrap support (Fig. 4), but also enabled the successful downstream identification by ARMS (Fig. 5). From an evolutionary viewpoint, the proximity of the Arabic *M. peregrina* with the Indian *M. oleifera* allows for interesting insights of the evolutionary and biogeographic dynamics of the genus, extending previous finding^15^ on the clear separation of African and Asian *Moringa* species. In fact, the Arabic *M. peregrina* lives up to its name (*peregrina* for pilgrim), since it turns out to be intermediate not only in terms of geography, but in terms of phylogeny as well.

The amplified refractory mutation system (ARMS) is a duplex-based PCR strategy to differentiate closely related species, and has been successfully employed to detect adulterations in bamboo teas^44^, authenticate the “superfood” Goji^45^, or reveal the threat to Peruvian *kiwicha* (*Amaranthus caudatus* L.) by seeds imported from the US^58^. In those studies, the barcoding markers *rbcLa* (bamboo), and *psbA-trnH-*igs (Goji and Amaranth) were useful. To the best of our knowledge, the *ycf1b* marker region has not been employed for ARMS-based diagnostics, but turned out to harbour great potential, as demonstrated by our present study. One major advantage of the *ycf1b* region is its considerable length and the absence of repetitive regions, yielding higher possibilities for informative nucleotide substitutions compared to other markers. On the other hand, the length can also be a challenge for authentication studies, when commercial products are processed to an extent that only fragmented DNA can be extracted, such that the required amplification of long (900 base pairs) fragment would not be possible. While this limitation could be fixed by choosing shorter regions spanning the informative domains, in the case of *Moringa*, the quality of extracted DNA from commercial products (also from powders) was sufficient to amplify the entire *ycf1b* fragment. By designing two complementary ARMS primers, we were able to generate the diagnostic double band either, when *M. oleifera* was present, or, when the specimen contained *Moringa* species other than *M. oleifera*. A positive readout (detection of a diagnostic band) is always more trustworthy as readout of an assay as compared to a negative readout (which might also be caused by failure of the assay per se). Thus, testing a given sample by both approaches, will inevitably produce a clear result. In addition, the ARMS approach has the advantage of an innate control consisting in the full-length band of the respective marker (in our case, *ycf1b*), further safeguarding against false-negative results that might be caused by a suboptimal PCR. Since the identity of commercial samples is difficult to establish, and the declaration of commercial *Moringa* products are anything else than trustworthy, we used our taxonomically authenticated reference plants to validate this test. To our opinion, the reliability of any diagnostic assay stands or falls with the use of authenticated references, an aspect that should become a standard in the field.

The simplicity of the sequencing-free ARMS assay represents, also, its weak point: As long as *Moringa* is traded as single product, this assay can both, validate the presence of *Moringa oleifera*, or exclude the presence of other *Moringa* species depending on the diagnostic primer used. However, if the commercial product contains a mixture of different plants, the resulting patterns will quickly turn difficult to interpret^59^. Here, one could combine the amplification of the barcode Next-Generation Sequencing, focusing on the informative signatures^60^, which would also add quantitative data on the composition of the sample. A further development would be the use of real-time qPCR, in order to quantify amplicons of interest^61^.

The rapid growth of the European market for *Moringa* provides very attractive opportunities for African producers, which not only is home to many members of the genus, but also allows for the cultivation of *M. oleifera*, the only species, which can be legally traded in Europe. In fact, these opportunities have been recognised and Moringa production is actively promoted, also by developmental programmes, for instance in South Africa (for a recent review see^62^). To expect that farm small-holders and villagers trying to develop Moringa as new source of income develop a consciousness for the subtleties of plant taxonomy, would be naïve. Thus, a robust, rapid, and sequencing-free authentication assay is urgently needed. This assay can be used for different purposes – on the one hand, it can serve importers to safeguard their own business as well as the consumers to import non-eligible Moringa products. Alternatively, as long as importers have not developed the needed sensitivity to the issue, it can also serve European authorities in charge of food safety to monitor traded Moringa products. However, and this application may be even more important, the same assay system could be used to ensure that the producers in Africa are provided with authentic seed material.

## Methods

### Reference plants and commercial samples

A combination of fresh and dried leaf samples from different *Moringa* species (Table 1) served as reference material in this study. Different accessions of *M. oleifera*, *M. drouhardii*, *M. hildebrandtii* Engl*., M. peregrina* (Forssk.) Fiori, *M. stenopetala*, and *M. ovalifolia* Dinter & Berger are grown and maintained at the Botanical Garden of Karlsruhe Institute of Technology (KIT), Karlsruhe, Germany. Leaves were either collected freshly from the plants grown at the Botanical Garden or were obtained (in dried form) from sources outside the Karlsruhe Institute of Technology. Additionally, *Carica papaya* L. and *C. x pentagona* (V.M.Badillo) V.M.Badillo (Mountain papaya), both grown in the Botanical Garden of Karlsruhe Institute of Technology, were selected as outgroups for the phylogenetic analysis. Twelve commercial samples, in different forms, and from different commercial sources, were included into the analysis (Table 2).

**Table 1:** List of reference plants of Moringa and Carica used for the current study

taxon	voucher ID	part used	Source of plant	*GenBank psbA-trnH* igs	*GenBank ycf1b*
*M. oleifera* Lam.	8468	entire plant	Rühlemanns (GE)	MT916786	MT916816
*M. oleifera* Lam.	9069	entire plant	Moringa Garden, Tenerife (SP)	MT916787	MT916817
*M. oleifera* Lam.	9140	entire plant, red buds	Moringa Garden, Tenerife (SP)	MT916789	MT916818
*M. oleifera* Lam.	9140	entire plant, white buds	Moringa Garden, Tenerife (SP)	MT916788	MT916827
*M. oleifera* Lam.	9007	entire plant	Asklepios Seeds (GE)	–	MT916819
*M. oleifera* Lam.	9157	dry leaf	Isiolo, Kenia (KE)	–	MT916832
*M. oleifera* Lam.	9161	dry leaf	www.vitalundfitmit100.de (GE)	MT916790	–
*M. oleifera* Lam.	9174	dry leaf, red buds	Moringa Garden, Tenerife (SP)	MT916791	MT916838
*M. oleifera* Lam.	9175	dry leaf, white buds	Moringa Garden, Tenerife (SP)	–	MT916839
*M. oleifera* Lam.	9259	dry leaf	Varanasi, Uttar Pradesh (IN)	–	MT916840
*M. oleifera* Lam.	9260	dry leaf	Varanasi, Uttar Pradesh (IN)	MT916792	MT916841
*M. oleifera* Lam.	9261	dry leaf	Varanasi, Uttar Pradesh (IN)	MT916794	MT916842
*M. drouhardii* Jum.	8908	entire plant	BGU Saarbrücken (GE)	MT916797	MT916820
*M. drouhardii* Jum.	9159	dry leaf	BGU Heidelberg (GE)	-	MT916829
*M. drouhardii* Jum.	9158	dry leaf	BGU Heidelberg (GE)	MT916798	MT916835
*M. stenopetala* (Baker f.) Cufod.	8698	entire plant	Rühlemanns (GE)	MT916803	MT916821
*M. stenopetala* (Baker f.) Cufod.	9008	entire plant	Asklepios Seeds (GE)	MT916805	MT916823
*M. stenopetala* (Baker f.) Cufod.	9155	dry leaf	Rainer Martin, Bielefeld Kenia, Nördl. Isiolo	MT916805	MT916831
*M. stenopetala* (Baker f.) Cufod.	9156	dry leaf	Rainer Martin, Bielefeld	MT916806	MT916833
*M. stenopetala* (Baker f.) Cufod.	9173	dry leaf	Moringa Garden, Tenerife (SP)	MT916807	MT916837
*M. hildebrandtii* Engl.	9153	dry leaf	BGU Heidelberg (GE)	MT916799	MT916822
*M. hildebrandtii* Engl.	9172	dry leaf	Moringa Garden, Tenerife (SP)	MT916800	MT916836
*M. ovalifolia* Dinter & A.Berger (*)	9038	entire plant	Bonsai-shopping (GE)	MT916801	MT916824
*M. ovalifolia* Dinter & A.Berger (*)	9149	entire plant	Tropica (GE)	MT916802	MT916825
*M. peregrina* (Forssk.) Fiori	9147	entire plant	BGU Bonn (GE)	MT916795	MT916826
*M. peregrina* (Forssk.) Fiori	9154	dry leaf	Moringa Garden, Tenerife (SP)	MT916796	MT916830
*M. peregrina* (Forssk.) Fiori	9160	dry leaf	BGU Bonn (GE)	–	MT916834
*M. peregrina* (Forssk.) Fiori	9163	entire plant	RBG Kew (UK)	–	MT916843
*C. papaya L*.	5717	entire plant, female	BG KIT (GE)	–	MT916844
*C*. x *pentagona*	8892	entire plant	BGU Saarbrücken (GE)	–	MT916845

(*) has been received as *Moringa ovalifolia*, but morphological and molecular analysis indicates that the identity is *M. oleifera*.

**Table 2:** List of commercial products of *Moringa* used for the current study

taxon	voucher ID	Appearance of product	*psbA-trnH* igs	*ycf1b*
*Moringa spec*.	2568	Herbal tea blend	MT916782.1	MT916814.1
*Moringa spec*.	3464	Herbal tea blend	MT916783.1	*–*
*Moringa spec*.	3465	Herbal tea blend	MT916784.1	MT916815.1
*Moringa spec*.	3466	Tea, leaf cut dried	MT916776.1	MT916809.1
*Moringa spec*.	3468	Herbal tea, leaf cut dried	MT916775.1	MT916808.1
*Moringa spec*.	3469	Powder	MT916780.1	MT916812.1
*Moringa spec*.	9312	Tea, leaf cut dried	MT916777.1	*–*
*Moringa spec*.	9313	Tea, leaf cut dried	MT916778.1	MT916810.1
*Moringa spec*.	9315	Tea, leaf cut dried	MT916779.1	MT916811.1
*Moringa spec*.	9316	Powder	MT916793.1	MT916813.1
*Moringa spec*.	9317	Tea as powder	MT916781.1	MT916828.1
*Moringa spec*.	9320	Tea, leaf cut dried	MT916785.1	-

### Morphological characterisation

To identify the anatomical and morphological structures of *Moringa* commercial products, we used leaves from the reference plants. We had first verified the identity of these references by appropriate literature and determination keys^15,63–69^.

To get more detailed anatomical and morphological insight of the reference plants and the commercial products of *Moringa*, the leaves were inspected and compared by stereo (S6D, Leica) and bright-field microscopy (DM750, Leica). To ensure comparability, we chose leaflets from the terminal end of the main axis from the plants grown at the Botanical Garden of the KIT. In case of the dried leaves received from other sources, order and position of leaves were unknown. For macro-morphological analysis, we recorded the entire fresh or dried pinnate leaves of the respective specimen with a Nikon COOLPIX S8000 camera. If not stated otherwise, we examined entire leaves by stereomicroscopy at a magnification of 6.3 x for both, reference plants and commercial products. If the leaves were too large for microscopy, we split them with a razor blade, imaging the parts separately, and then reassembled the partial images. In case of powders or leaf fragments used in tea blends, we used a magnification of 25x. We prepared hand-cuts from the fresh the *Moringa* leaves. For the powder, or leaf fragments in commercial products, we bleached with chloral hydrate (60% v/v). Herefore, we placed the specimens on a glass slide along with a few drops of chloral hydrate, and heated for a few seconds using a Bunsen burner under a fume hood before viewing by bright-field microscopy at a magnification of 400 x. We analysed the adaxial and the abaxial faces of the bifacial leaves separately. In case of commercial leaf powder, several fragments of the powder were analysed to reduce sampling bias. In order to be able to assign fragments from trade products to a species, characteristic cell layers and diagnostic features were screened including the shape of the epidermis cells, organisation of guard-cell complexes, accessory cells, idioblasts, raphids, and trichomes. If the idioblasts were not visible, we cut a cross section. We visualised calcium oxalate crystals under crossed polarising filters. Images were recorded by a digital camera (SN 40110022 EC3, Leica, Bensheim) installed on the microscope.

### DNA extraction and PCR

DNA from fresh leaves of reference plants (using 60 mg of starting material) and commercial products (using 30 mg of starting material) was isolated using the Invisorb® Spin Plant Mini Kit (Stratec Biomedical AG), following the instructions of the manufacturer. Quality and quantity of isolated DNA were evaluated by spectrophotometry (Nanodrop, Peqlab), and DNA concentration was diluted to 50 ng / µl to be used as template in PCR.

We amplified the barcoding marker sequences in a reaction volume of 30 µl, containing 20.4 µl nuclease free water (Lonza, Biozym), 3 µl of 10-fold Thermopol Buffer (New England Biolabs), 3 µl of 10 mg / ml bovine serum albumin, and 0.6 µl of 1.5 mM dNTPs (New England Biolabs). We used 100–150 ng of DNA template, 0.6 µl of 10 µM of each, forward and reverse primers (see primer list, Table 3), and 0.3 µl of Taq polymerase (New England Biolabs) for a single reaction.

**Table 3:** Oligonucleotide primers used in the current study

Name	5ʹ → 3ʹ sequence	T_m_	Target	Design
psbA^*u*^	GTTATGCATGAACGTAATGCTC	61.0 °C	*psbA-trnH* intergenic spacer	Sang et al. ^70^
trnH^*u*^	CGCGCATGGTGGATTCACAATCC	76.0 °C		Tate et al. ^71^
ycf1b_fw^*u*^	TCTCGACGAAAATCAGATTGTTG	69.9 °C	*ycf1b* coding region	Dong et al. ^39^
yfc1b_rv^*u*^	ATACATGTCCAAAGTGATGGAAA	59,8 °C		
ycf1b_Mo_ ARMS_rv^*d*^	CTTTGATCGTTGGGATCAGTCAG	66.7 °C	diagnostic SNP in *M oleifera ycf1b*	current study
ycf1b_NoMo_ ARMS_rv^*d*^	GTTTGAATTTTCTTGAACGAATCTG	63,6 °C	diagnostic SNP in *Moringa* species other than *M. oleifera*	current study

^*u*^ universal.

^*d*^ diagnostic.

The plastidic *psbA-trnH* igs region was amplified by initial denaturation at 95 °C for 2 min; following 33 cycles at 94 °C for 1 min, 56 °C for 30 s, 68 °C for 45 s; ending with an extension of 68 °C for 5 min. For the plastidic marker *ycf1b*, the initial denaturation at 95 °C for 2 min was followed by 35 cycles at 94 °C for 30 s, 52 °C for 40 s, 68 °C for 1 min, ending with an extension of 68 °C for 10 min.

After agarose gel electrophoresis, using NEEO ultra-quality agarose (Carl Roth, Karlsruhe, Germany), we visualised the amplicons using either SYBRsafe (Invitrogen, Thermo Fisher Scientific Germany) or Midori green Xtra (Nippon Genetics Europe GmbH) and blue light excitation. The fragment size was determined using a 100-bp size standard (New England Biolabs). We then purified the amplicons using the MSB® Spin PCRapace kit (Stratec) and subsequently obtained the sequences from a commercial provider (Macrogen Europe, Netherlands, or GATC, Germany).

We assessed the quality of the obtained sequences with the software FinchTV (Version 1.4.0). To get a more robust result, we generated sequences from two directions of the amplicon for each accession, merging the forward read with the reverse complemented read from the opposite direction.

### Phylogenetic analysis

We aligned the sequences and constructed their phylogenetic relationship using the freeware MEGA7 (Version 7.0.14) with the integrated Tree Explorer. After alignment using the Muscle algorithm, we trimmed the alignments to the first nucleotide downstream of the forward primer, and the last nucleotide preceding the reverse primer. We inferred evolutionary relationships by using the neighbour-joining algorithm with a bootstrap value that was based on 1000 replicates. As outgroups to evaluate the *Moringa* dataset, we selected *Carica papaya* and *Carica x pentagona*.

### ARMS diagnostics

A single nucleotide difference in the *ycf1b* sequences of *Moringa oleifera* and the other members of the genus allowed designing diagnostic primers to discriminate *M. oleifera* clearly from all related species in a one-step duplex-PCR protocol. At position 226 of the multiple sequence alignment for *ycf1b*, *M. oleifera* harbours a guanine, while all other *Moringa* species show a thymine at this site. We, therefore, placed this nucleotide at the 3′-end of the diagnostic primer, and exchanged an additional nucleotide at the third position of the 3′-end, to reduce the primer affinity to this target region in other *Moringa* species. Choosing this design, the diagnostic primer (ycf1b_Mo_ARMS_rv) should only be able to bind to amplicons from *M. oleifera*. As complementary strategy, a second diagnostic primer (ycf1b_NoMo_ARMS_rv) was designed that should only bind to the ycf1b region from other *Moringa* species, but not to those from *M. oleifera*. In both cases, the diagnostic primer would amplify an additional fragment that would appear during subsequent agarose gel electrophoresis in addition to the larger band from the universal ycf1b_fw and ycf1b_rv primers (Table 3).

### Reporting summary

Further information on research design is available in the Nature Research Reporting Summary linked to this article.

### Supplementary information

Supplementary Figures

Reporting summary

## Data Availability

Dataset – *ycf1b* sequences. https://www.ncbi.nlm.nih.gov/popset?DbFrom=nuccore&Cmd=Link&LinkName=nuccore_popset&IdsFromResult=1922723560. Dataset – *psbA-trnH igs* sequences. https://www.ncbi.nlm.nih.gov/popset?DbFrom=nuccore&Cmd=Link&LinkName=nuccore_popset&IdsFromResult=1922723306.
