# Supplementary material for: Monitoring Indian “Superfood” Moringa oleifera Lam. – species-specific PCR-fingerprint-based authentication for more consumer safety
Source: NPJ Sci Food. 2024 Apr 13;8:21. doi: 10.1038/s41538-024-00264-z (PMC11016095; doi:10.1038/s41538-024-00264-z)
Supplement: Supplementary file 1 — Supplementary Figures [file 41538_2024_264_MOESM1_ESM.pdf]

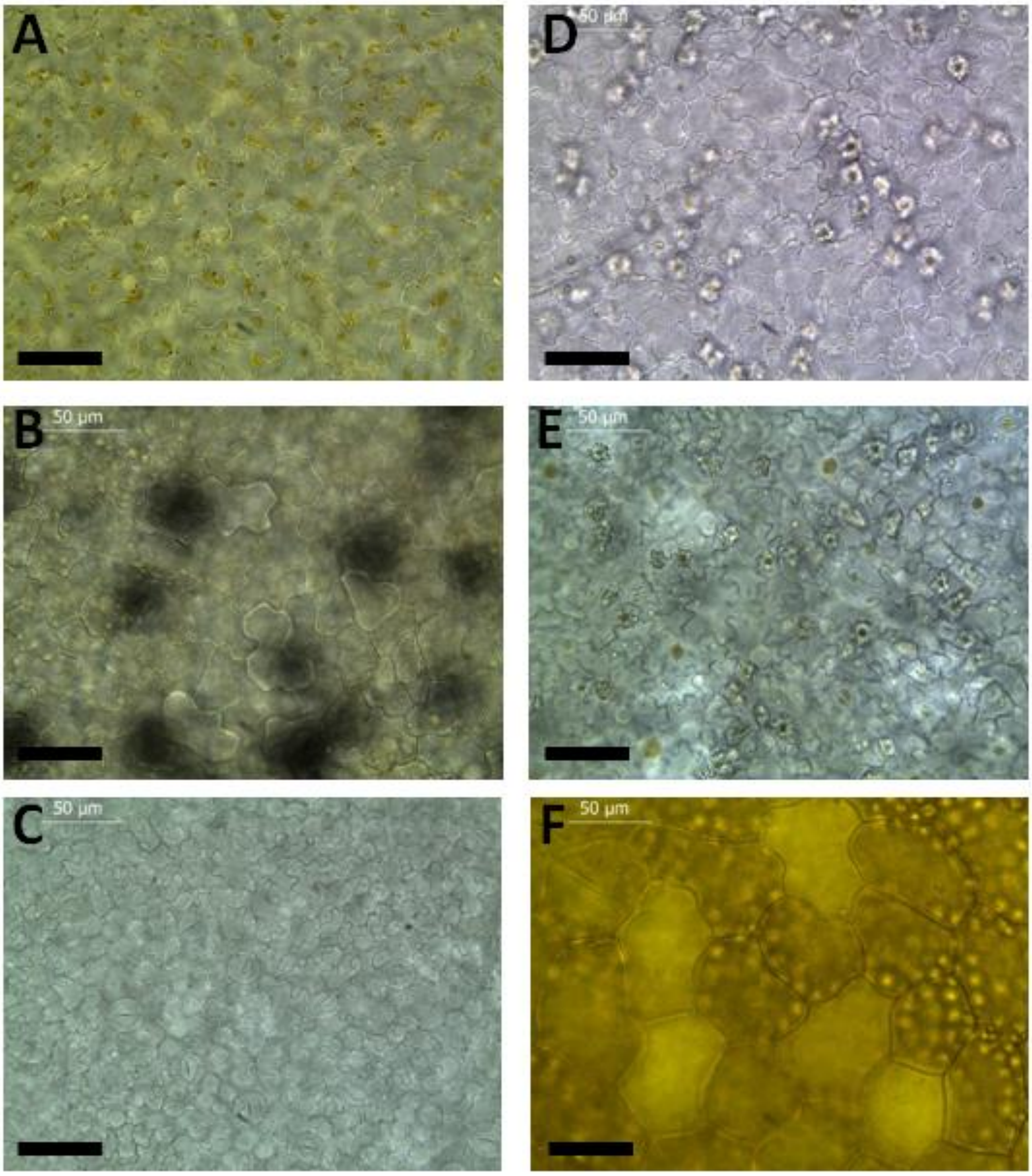

Supplementary Figure 1: Morphological characterisation of leaf epidermal cell layer of *Moringa*.

The images were taken at a magnification of 400x. (Scale bar, 50 µm). **(*)** received as *M. ovalifolia*, by reevaluation determined as *M. oleifera*.

(A) *Moringa oleifera,* (B) *M. ovalifolia***(*)***,* (C) *M. peregrina,* (D) *M. stenopetala,* (E) *M. drouhardii and* (F) *M. hildebrandtii.*

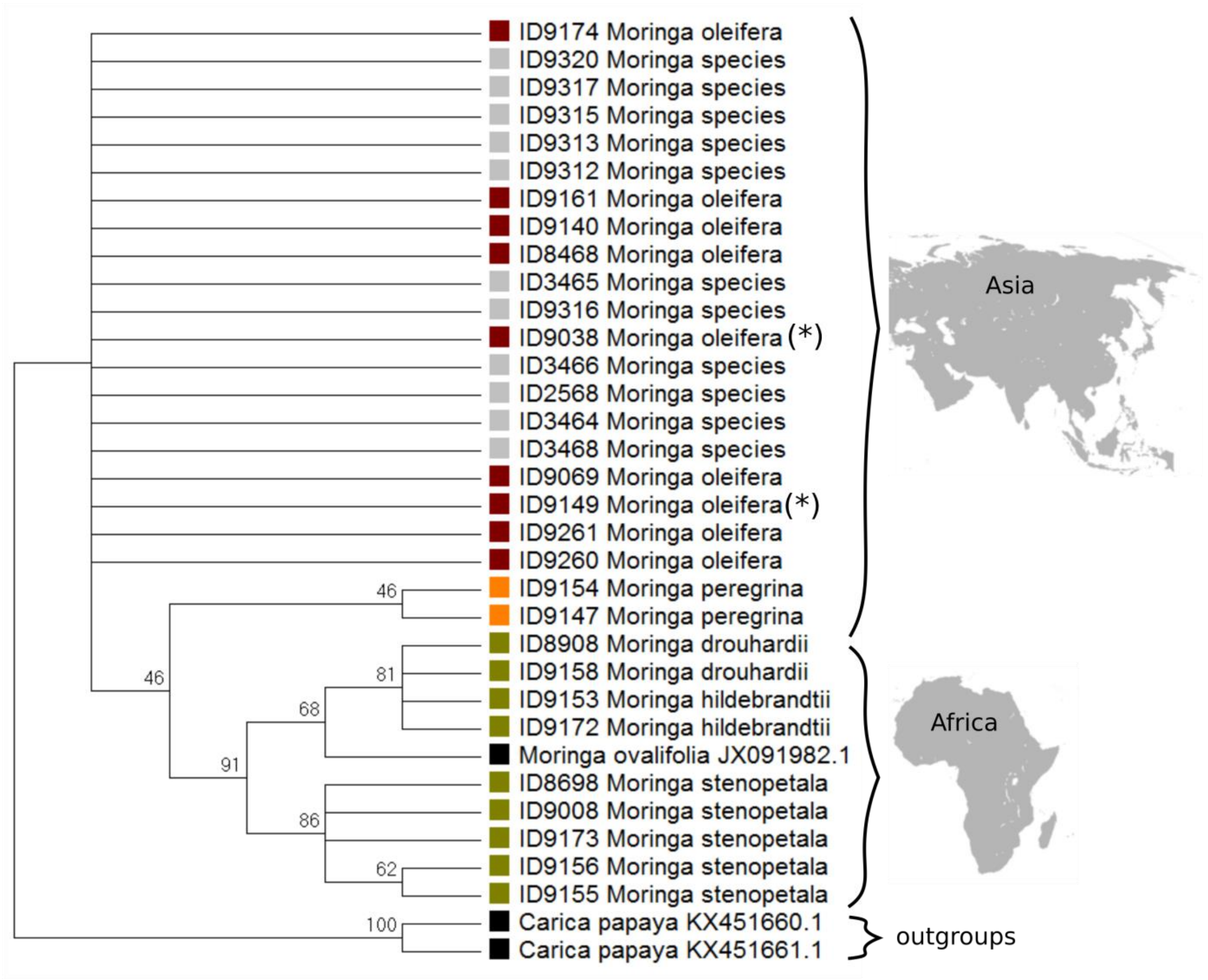

Supplementary Figure 2: Phylogenetic tree based on *psbA-trnH* igs spacer region inferred by Neighbour Joining. Reference plants are represented as colored squares, with red for Indian *M. oleifera*, yellow for African *Moringa* species (including *M. hildebrandtii*, *M. stenopetala*, and *M. drouhardii*) and orange for *M. peregrina*. Commercial products are displayed with grey squares. The outgroup *Carica papaya* is represented as black squares. The internal ID of the Botanical Garden of the KIT (see also table 1) are given next to the species name. The numbers on the branches represent the bootstrap values estimated from 1000 replications. The geographic regions are not drawn to scale. **(*)** received as *M. ovalifolia*, by reevaluation determined as *M. oleifera.*
